# Distortion correction in TSE near titanium implants at 0.55 T using reversed frequency-encoding and model-based reconstruction

**DOI:** 10.1007/s10334-025-01315-6

**Published:** 2025-12-22

**Authors:** Bochao Li, Nam G. Lee, Daehyun Yoon, Kübra Keskin, Alexander R. Toews, Jay Acharya, Jordan S. Gross, Brian A. Hargreaves, Krishna S. Nayak

**Affiliations:** 1 Alfred E. Mann Department of Biomedical Engineering, University of Southern California, 3740 McClintock Ave, EEB 416, Los Angeles, CA 90089-2564 USA; 2 Radiology and Biomedical Imaging, University of California San Francisco, San Francisco, CA USA; 3 Ming Hsieh Department of Electrical and Computer Engineering, University of Southern California, Los Angeles, CA USA; 4 Electrical Engineering, Stanford University, Stanford, CA USA; 5 Diagnostic Radiology, University of California Los Angeles, Los Angeles, CA USA; 6 Radiology, Stanford University, Stanford, CA USA

**Keywords:** Metallic implant, Gradient nonlinearity, Off-resonance, Mid-field MRI, 0.55 Tesla MRI

## Abstract

**Objectives:**

To develop a method for imaging near titanium implants at 0.55 T, which enables the use of a low readout bandwidth for higher SNR while reducing in-plane geometric distortions.

**Materials and methods:**

A pair of turbo spin echo (**TSE**) data with opposite frequency-encoding directions is acquired. For each frequency direction, a gradient nonlinearity (**GNL**)-corrected image is reconstructed with a model-based iterative reconstruction incorporating GNL. A susceptibility-induced displacement map along the readout direction is estimated from two GNL-corrected images. A single final image is reconstructed with the model-based reconstruction incorporating both GNL and metal-induced displacement fields using both k-space acquisitions. The proposed method is compared against TSE with view angle tilting (**VAT**) and slice encoding for metal artifact correction (**SEMAC**).

**Results:**

The proposed reconstruction method maintains spatial resolution compared to current correction techniques. Unlike image-domain correction, VAT, and SEMAC, it does not introduce spatial blurring at equivalent bandwidths. It is feasible to reduce distortion due to off-resonance at a low readout bandwidth, resulting in higher apparent SNR (1.4–1.6-fold) without blurring under current imaging settings. In phantoms, and in patients with total hip arthroplasty and spinal fusion, the proposed method provides clearer delineation of tissues compared to conventional methods.

**Discussion:**

The proposed GNL and off-resonance distortion correction method for imaging near metal at 0.55 T enables the use of low readout bandwidth, providing SNR improvements without the blurring typically associated with low-bandwidth VAT.

**Supplementary Information:**

The online version contains supplementary material available at 10.1007/s10334-025-01315-6.

## Introduction

Orthopedic metallic implants are widely used to improve the quality of life for people who experience injury or degeneration. The most common procedures include knee replacements, hip replacements, and spinal fusion procedures [1]. Titanium alloy is one of the common materials for such implants due to biocompatibility and appropriate mechanical characteristics [2]. MRI, when successful, provides detailed soft-tissue contrast around implants, making it an ideal tool for post-surgical assessment, longitudinal follow-up, and planning for revision procedures [3, 4]. However, the susceptibility difference between metallic implants and surrounding tissues induces off-resonance effects, leading to signal displacement artifacts, particularly along the frequency-encoding direction, obscuring critical diagnostic information [5, 6]. Multispectral imaging methods, such as slice encoding for metal artifact correction (**SEMAC**) [7] and multi-acquisition with variable-resonances image combination selective (**MAVRIC-SL**) [8], significantly reduce off-resonance artifacts by acquiring multiple spectral images combined into composite images. However, both methods require longer scan times due to the need for a larger number of spectral acquisitions.

View angle tilting (**VAT**)[9] is commonly used to mitigate in-plane distortions without prolonging scan time along with turbo spin echo (**TSE**), but results in (in-plane) spatial blurring, especially with lower bandwidths. Low-field MRI (≤ 0.55 T) inherently reduces metal artifacts due to weaker off-resonance [10–16], but the reduced SNR requires compensation to detect subtle signal changes such as osteolysis and synovitis in postoperative hip [17] and spine [18]. Increased SNR at 0.55 T can be achieved with multiple averages, but this requires a longer scan time. Alternatively, lower bandwidths can be used to enhance SNR, but this strategy aggravates frequency-encoding (readout) displacement artifacts, which result in significant blurring when used with VAT.

Metal-artifact mitigation strategies based on two images acquired with reversed readout gradients, referred to as the reversed frequency-encoding approach, have shown promise in distortion correction at higher fields [19–21]. However, using reversed frequency-encoding is challenged by the extreme spatial derivative of B_0_-dependent off-resonance, $\frac{\partial \Delta f\left(x\right)}{\partial x}$, at high fields [6, 22]. At 0.55 T, the off-resonance frequency gradient is 5.5 and 2.7 times smaller compared to 3 T and 1.5 T, respectively, presenting an opportunity to revisit this correction technique for metal-induced distortions at low fields. In addition, gradient nonlinearity (**GNL**), causing the geometric distortion in images, can interfere with the diagnostic value. Model-based reconstruction, including GNL, is appealing in preserving resolution [23].

In this study, we investigate a VAT-free metal artifact reduction technique at 0.55 T. With a pair of TSE datasets with opposite frequency-encoding directions [22], denoted as reversed-encoding TSE (**RE-TSE**), we estimate a displacement map (readout direction only) due to metal-induced off-resonance using 3D cubic b-spline basis sets [19, 20]. Inspired by previous work by Tao et al.[23], we propose a model-based image reconstruction framework to mitigate both displacement fields induced by gradient nonlinearity and metal-induced off-resonance during image reconstruction. Incorporating spatial displacement fields using a type-1 NUFFT [24] in a forward model, this framework avoids spatial smoothing caused by image-based geometric correction methods used in a vendor-provided GNL correction routine, retaining sharpness in distortion-corrected images. Gradient delay causes different phases in the two opposite TSE acquisitions [25], which we employ a phase-constrained framework to mitigate [26, 27]. Due to less number of unknowns recovered from the same number of measurements [28], a single corrected image can preserve high SNR reconstructed from two TSE acquisitions. We demonstrate distortion-corrected high-resolution imaging around metallic implants, enhancing SNR at 0.55 T.

## Methods

### Signal modeling

The susceptibility differences between metal implants and surrounding tissue cause significant off-resonance (∆ƒ). This off-resonance will cause distortions in both the slice and readout directions in 2D spin echo (**SE**) and TSE imaging. The presence of gradient nonlinearity causes geometric distortions both in the phase-encoding and readout directions. Considering off-resonance distortion in the readout direction only and in-plane GNL distortions, the forward signal model for k-space data of the *c*th coil at the *i*th phase-encoding step (out of a total of ${N}_{i}$ steps) can be expressed as:

1 $${d}_{c,i}\left({k}_{x}\left(t\right),{k}_{y,i}\right)=\int m\left(\mathbf{r}\right){S}_{c}\left(\mathbf{r}\right){e}^{+j\left({k}_{x}\left(t\right)\left(x+\frac{2\pi \Delta f\left(\mathbf{r}\right)}{\gamma {G}_{r}}+\Delta x\left(\mathbf{r}\right)\right)+{k}_{y,i}\left(y+\Delta y\left(\mathbf{r}\right)\right)\right)}d\mathbf{r}+{n}_{c}\left({k}_{x}\left(t\right),{k}_{y,i}\right)$$

where $m\left(\mathbf{r}\right)$ is the image to be recovered, ${S}_{c}\left(\mathbf{r}\right)$ is the receive coil sensitivity at position $\mathbf{r}$ of the *c*th coil, $\Delta f\left(\mathbf{r}\right)$ is the metal-induced off-resonance (in Hertz), ${G}_{r}$ is the amplitude of the readout gradient (in mT/m), ${k}_{x}\left(t\right)$ and ${k}_{y,i}$ are the k-space positions, $\Delta x\left(\mathbf{r}\right)$ and $\Delta y\left(\mathbf{r}\right)$ denote the spatially varying displacements due to GNL in the readout and phase-encoding directions (in meters), and ${n}_{c}$ denotes the measurement noise. According to Tao et al.[23], discretizing in time with ${N}_{k}$ time steps and in space with N spatial locations leads to

2 $${d}_{c}\left({k}_{x}\left({t}_{\kappa }\right),{k}_{y,i}\right)=\sum_{\rho =1}^{N}\begin{array}{c}m\left({\mathbf{r}}_{\rho }\right){S}_{c}\left({\mathbf{r}}_{\rho }\right){e}^{+j\left({k}_{x}\left({t}_{\kappa }\right)\left({x}_{\rho }+\frac{2\pi \Delta f\left({\mathbf{r}}_{\rho }\right)}{\gamma {G}_{r}}+\Delta x\left({\mathbf{r}}_{\rho }\right)\right)+{k}_{y,i}\left({y}_{\rho }+\Delta y\left({\mathbf{r}}_{\rho }\right)\right)\right)}\\ +{n}_{c}\left({k}_{x}\left({t}_{\kappa }\right),{k}_{y,i}\right),\end{array}$$

where ${\mathbf{r}}_{\rho }$ is the spatial location of the center of the ρ th pixel. In matrix form, Eq. 2 can be expressed as

3 $${\mathbf{d}}_{c}=\left[\begin{array}{c}{\mathbf{d}}_{c,1}\\ \vdots \\ {\mathbf{d}}_{c,{N}_{i}}\end{array}\right]=\mathbf{E}{\mathbf{S}}_{c}\mathbf{m}+{\mathbf{n}}_{c}$$

where ${\mathbf{d}}_{c,i}={\left[{d}_{c}\left({k}_{x}\left({t}_{1}\right),{k}_{y,i}\right),\dots ,{d}_{c}\left({k}_{x}\left({t}_{{N}_{k}}\right),{k}_{y,i}\right)\right]}^{T}\in {\mathbb{C}}^{{N}_{k}}$,${\mathbf{S}}_{c}\in {\mathbb{C}}^{N\times N}$ is the diagonal matrix of coil sensitivities of the *c*th coil, and $\mathbf{E}\in {\mathbb{C}}^{{{N}_{i}N}_{k}\times N}$ represents the Fourier transform from nonuniform image space to uniform k-space with its element defined by

4 $${\mathbf{E}}_{(i,\kappa ),\rho }={e}^{+j\left({k}_{x}\left({t}_{\kappa }\right)\left({x}_{\rho }+\frac{2\pi \Delta f\left({\mathbf{r}}_{\rho }\right)}{\gamma {G}_{r}}+\Delta x\left({\mathbf{r}}_{\rho }\right)\right)+{k}_{y,i}\left({y}_{\rho }+\Delta y\left({\mathbf{r}}_{\rho }\right)\right)\right)}$$

### Experimental methods

All experiments were conducted on a 0.55 T whole-body system (prototype MAGNETOM Aera XQ; Siemens Healthineers; Forchheim, Germany) equipped with high-performance shielded gradients (45 mT/m amplitude, 200 T/m/s slew rate). A 6-channel body array (anterior) and an 18-channel spine array coil (posterior) were used for phantom studies and total hip arthroplasty (**THA**) subjects. A 16-channel head and neck coil along with one element of the spine coil were used for the cervical spine subject. All in vivo experiments were performed under a protocol approved by our Institutional Review Board, after obtaining written informed consent.

The following sequences were utilized—**RE-TSE:** a pair of turbo spin echo (**TSE**) datasets with opposite frequency-encoding directions, referred to as reversed-encoding TSE**; TSE-VAT**: TSE with view angle tilting**; SEMAC 6**: slice encoding for metal artifact correction[7] with six z-phase encodings. Supplementary Information Tables S1, S2, and S3 contain all imaging parameters**.**

#### Phantom experiments

To assess the performance of the proposed method in resolution preservation and artifact correction, an American College of Radiology (**ACR**) phantom [29] with a resolution insert was scanned with RE-TSE and TSE-VAT sequences with an in-plane resolution of 0.875 mm^2^. To evaluate the efficacy of off-resonance distortion correction, a hip implant phantom comprising a ceramic head and titanium stem immersed in water was scanned with RE-TSE and TSE-VAT sequences, each using high (401 Hz/Px) and low (200 Hz/Px) readout bandwidths. The orientation of this phantom relative to the main magnetic field was well matched to a typical orientation of an in vivo THA. A plastic grid was placed under the hip implant for the analysis of blurring and distortion. SEMAC 6 with a bandwidth of 401 Hz/Px was also acquired as a reference method.

#### In vivo experiments

Three patients with THA were scanned with RE-TSE and TSE-VAT sequences, each using two bandwidths: 401 Hz/Px and 150 Hz/Px. A high-bandwidth SEMAC 6 sequence (401 Hz/Px) was used as a reference. One subject with anterior cervical discectomy and fusion (**ACDF**) was scanned with a TSE sequence using two bandwidths (401 Hz/Px and 120 Hz/Px), a high-bandwidth TSE-VAT sequence (401 Hz/Px), and a low-bandwidth RE-TSE sequence (120 Hz/Px).

### Image reconstruction

**Fig. 1:**
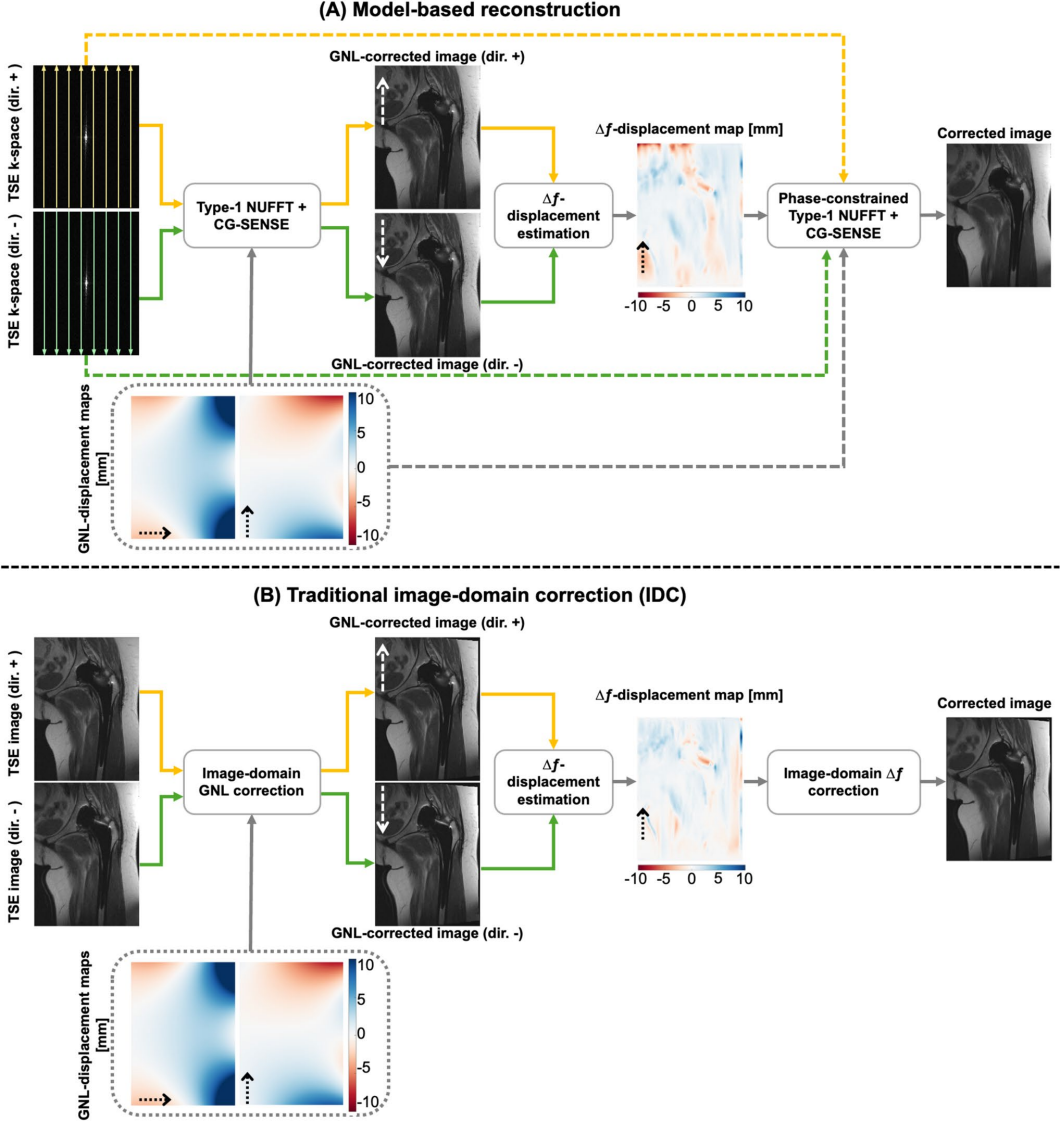
Flowchart of the **A** proposed distortion correction approach and **B** traditional image-domain correction (**IDC**). In the proposed method, each TSE dataset is separately reconstructed with Type-1 NUFFT-based CG-SENSE to correct geometric distortion induced by GNL. A displacement map due to off-resonance is estimated using both GNL-corrected images. A phase-constrained approach is combined with Type-1 NUFFT-based CG-SENSE to incorporate phase differences between two readout directions. A single image is reconstructed from two TSE datasets while simultaneously mitigating geometric distortions induced by GNL and off-resonance. Black arrow points the positive displacement direction in each displacement map

Figure 1A shows the RE-TSE reconstruction pipeline. A GNL-corrected image for each acquisition (indexed by *n*) of RE-TSE is reconstructed by solving the following cost function with the preconditioned conjugate gradient method (**PCG**):

5 $${\widehat{\mathbf{m}}}_{n}=\underset{{\mathbf{m}}_{n}\in {\mathbb{C}}^{N}}{\mathrm{argmin}}\sum_{c=1}^{{N}_{c}}{\Vert {\mathbf{E}}_{n}{\mathbf{S}}_{c,n}{\mathbf{m}}_{n}-{\mathbf{d}}_{c,n}\Vert }_{{{\ell}}_{2}}^{2} \text{ with } n=\mathrm{1,2},$$

where ${\mathbf{d}}_{c,n}$ is the k-space data of the *c*th coil,* n*th frequency-encoding direction, ${\mathbf{m}}_{n}$ is the *n*th GNL-corrected image, ${\mathbf{S}}_{c,n}$ is the coil sensitivity map for the *n*th TSE acquisition, and ${\mathbf{E}}_{n}$ is efficiently computed with the type-1 NUFFT [24, 30] where GNL displacement fields are calculated with spherical harmonic coefficients provided by a vendor. Coil sensitivity maps for each TSE acquisition were estimated by ESPIRiT [31].

A displacement map (due to off-resonance) is estimated from the pair of GNL-corrected magnitude images, ${\widehat{\mathbf{m}}}_{1}$ **and **${\widehat{\mathbf{m}}}_{2}$**,** modeled as a set of b-splines basis. 3.5 × 3.5 × 4 mm knot spacing is chosen similar to previous work [19, 20]. We use phase-constrained type-1 NUFFT-based CG-SENSE [32] reconstruction to resolve the phase difference between dual-polarity readout acquisitions. A single final distortion-corrected image $\mathbf{m}$ is estimated simultaneously from two readout acquisitions by minimizing the following cost function using PCG:

6 $$\widehat{\mathbf{m}}=\underset{\mathbf{m}\in {\mathbb{C}}^{N}}{\mathrm{argmin}}{\sum }_{n=1}^{2}\sum_{c=1}^{{N}_{c}}{\Vert {\mathbf{E}}_{n}{\mathbf{S}}_{c,n}\left({e}^{+j{\mathbf{P}}_{n}}\odot \mathbf{m}\right)-{\mathbf{d}}_{c,n}\Vert }_{{{\ell}}_{2}}^{2},$$

where the symbol ⊙ denotes the Hadamard product (i.e., element-wise multiplication), and ${\mathbf{P}}_{n}$ is the low-resolution phase image of the *n*th TSE acquisition, which is derived by taking the Fourier transform of Hamming-windowed 32 × 32 central k-space data.

In addition, a traditional image-domain correction (**IDC**) pipeline was implemented as one of the reference methods (Fig. 1B). Gradient-nonlinearity distortion was first corrected using the vendor-provided image-domain interpolation [33, 34], producing two GNL-corrected images acquired with opposite frequency-encoding directions. The off-resonance displacement field was then estimated from the spatial mismatch between these two GNL-corrected images, using the same reverse-encoding-based estimation procedure as in the proposed method. Finally, off-resonance correction was performed using the image-domain forward model described in Eq. 13 of Mullen et al. [19].

Image reconstructions were performed in MATLAB R2023b (MathWorks, Natick, MA) on a Linux workstation equipped with dual AMD EPYC 7502 processors (2.5 GHz) and 512 GB of RAM. Model-based reconstructions were implemented using the preconditioned conjugate gradients method (pcg.m). The algorithm stops either when it reaches the number of maximum iterations (e.g., 6 in this study) or when the relative residual $\frac{{\Vert y-Ax\Vert }_{{\ell}2}}{{\Vert y\Vert }_{{\ell}2}}$ fell below the tolerance of 1e − 3.

### Simulation

To evaluate the image quality of the proposed method with RE-TSE compared to TSE-VAT, a simulation was performed to quantitatively assess the correction on ∆ƒ-distortion and resultant image quality. The simulation framework [35] modeled spatially varying magnetic susceptibility and resulting ∆ƒ distributions near titanium hip implants with anatomical structures, while GNL effects were omitted. A noiseless TSE image without off-resonance effects served as the ground truth. Using the same imaging parameters as the experiments, images were generated for three imaging methods: TSE with two readout directions, TSE-VAT, and RE-TSE with the proposed reconstruction applied. Two readout bandwidths (400 Hz/Px and 200 Hz/Px) were simulated to investigate the effect of bandwidth on the image quality. To simulate realistic noise conditions, bivariate complex-valued Gaussian noise was added to achieve SNR levels of 15 and 9 (estimated from in vivo datasets) for the two bandwidths, respectively. Reconstructed images were compared against the ground-truth reference using error maps (voxel-wise difference from the reference) and the structural similarity index (**SSIM**) [36] with a Gaussian weighting filter (standard deviation = 1.5), and the root mean square error (**RMSE**), to quantify residual artifacts and structural fidelity.

### Image quality analysis

Line profiles were drawn from the phantom and in vivo images acquired with low readout bandwidths, since reducing readout bandwidth is the primary strategy for SNR improvement, which motivates the proposed method over TSE-VAT at 0.55 T. These profiles were used to illustrate the degree of spatial blurring observed with traditional image-domain correction (**IDC**) and TSE-VAT compared to the proposed model-based reconstruction.

Apparent SNR (**aSNR**) was computed for the hip implant phantom and all in vivo experiments. For each case, an identical region of interest (**ROI**) was selected across all methods to measure the mean signal intensity, while background regions were chosen to determine the noise standard deviation. For hip subjects, muscle tissue was chosen as the ROI. For cervical spine subjects, vertebrae were used as the ROI. The aSNR was then calculated as the ratio of the mean signal intensity to the noise standard deviation. The aSNR was calculated as the ratio of the mean signal intensity to the noise standard deviation [37]:

$$aSNR=\frac{S}{\sigma },$$

where S is the mean value of signal intensity over a ROI and σ is the standard deviation over an ROI in image background.

## Results

**Fig. 2:**
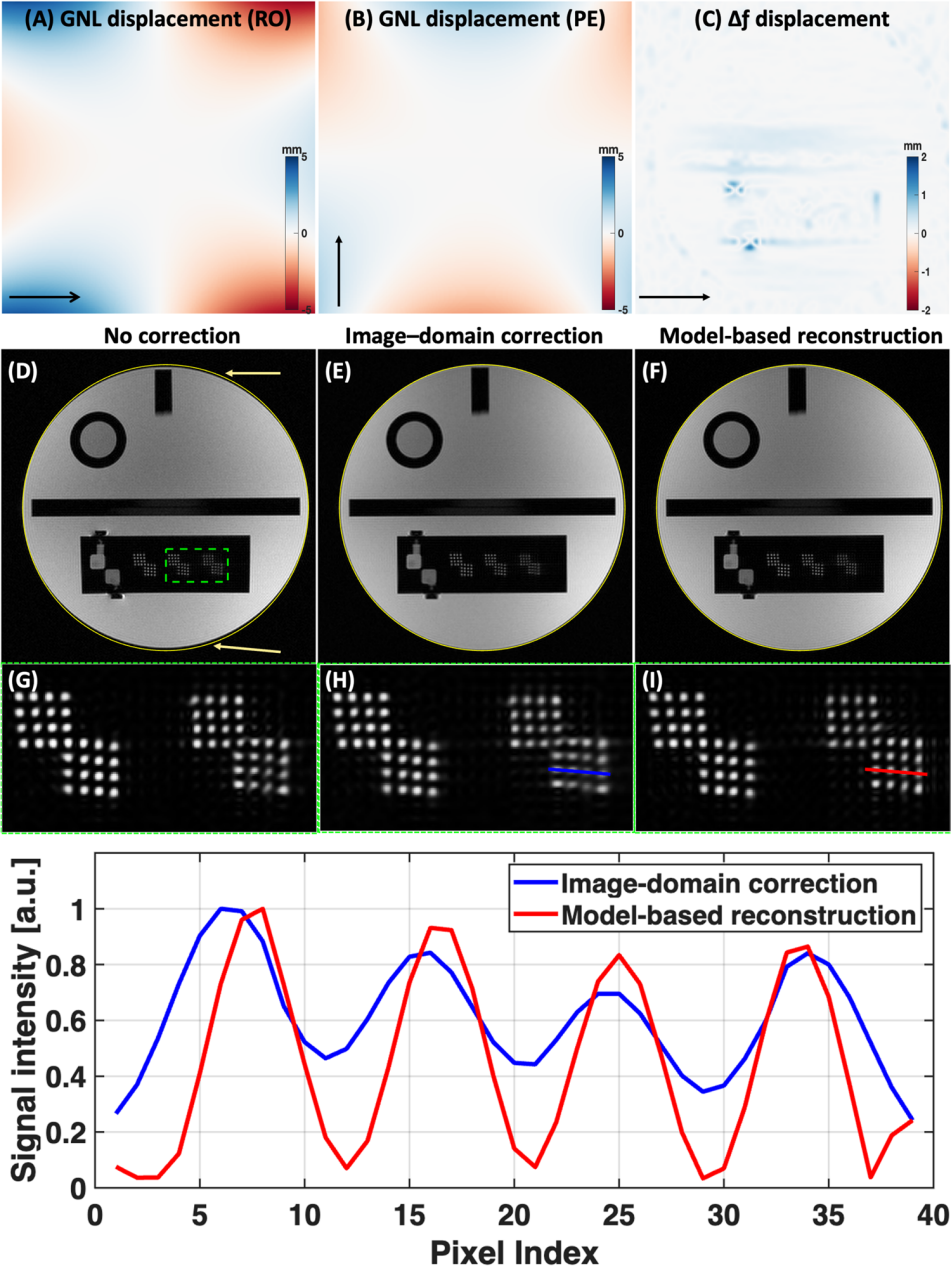
ACR phantom before and after GNL and ∆ƒ distortion correction (in-plane resolution = 0.875 mm^2^, 4 × sinc interpolation). **Row 1**: **A** and **B** GNL displacement map along readout and phase-encoding directions; **C** displacement map along readout direction due to off-resonance estimated from two GNL-corrected images during the model-based reconstruction**.** The displacement direction is labeled in each displacement map (black arrow). **Row 2**: **D** Image without GNL correction; **E** image-domain correction (IDC) for GNL and ∆ƒ; **F** proposed model-based reconstruction. **Row 3**: **G–I** magnified insets from **(D–F)**. **Row 4:** normalized line profile after image-domain correction and model-based reconstruction. The position of line profile is indicated with red markers

Figure 2 demonstrates the ability of the proposed Type-1 NUFFT-based CG-SENSE reconstruction in preserving spatial resolution after correcting GNL and off-resonance artifacts. Both the image-based method and proposed method show effective GNL correction preserving geometric accuracy in yellow dashed circles (the outline of the phantom) relative to the non-corrected image in (D). The sharper line profiles further illustrate that the proposed model-based reconstruction reduces the smoothing effect observed with IDC, thereby better preserving the spatial detail.

**Fig. 3:**
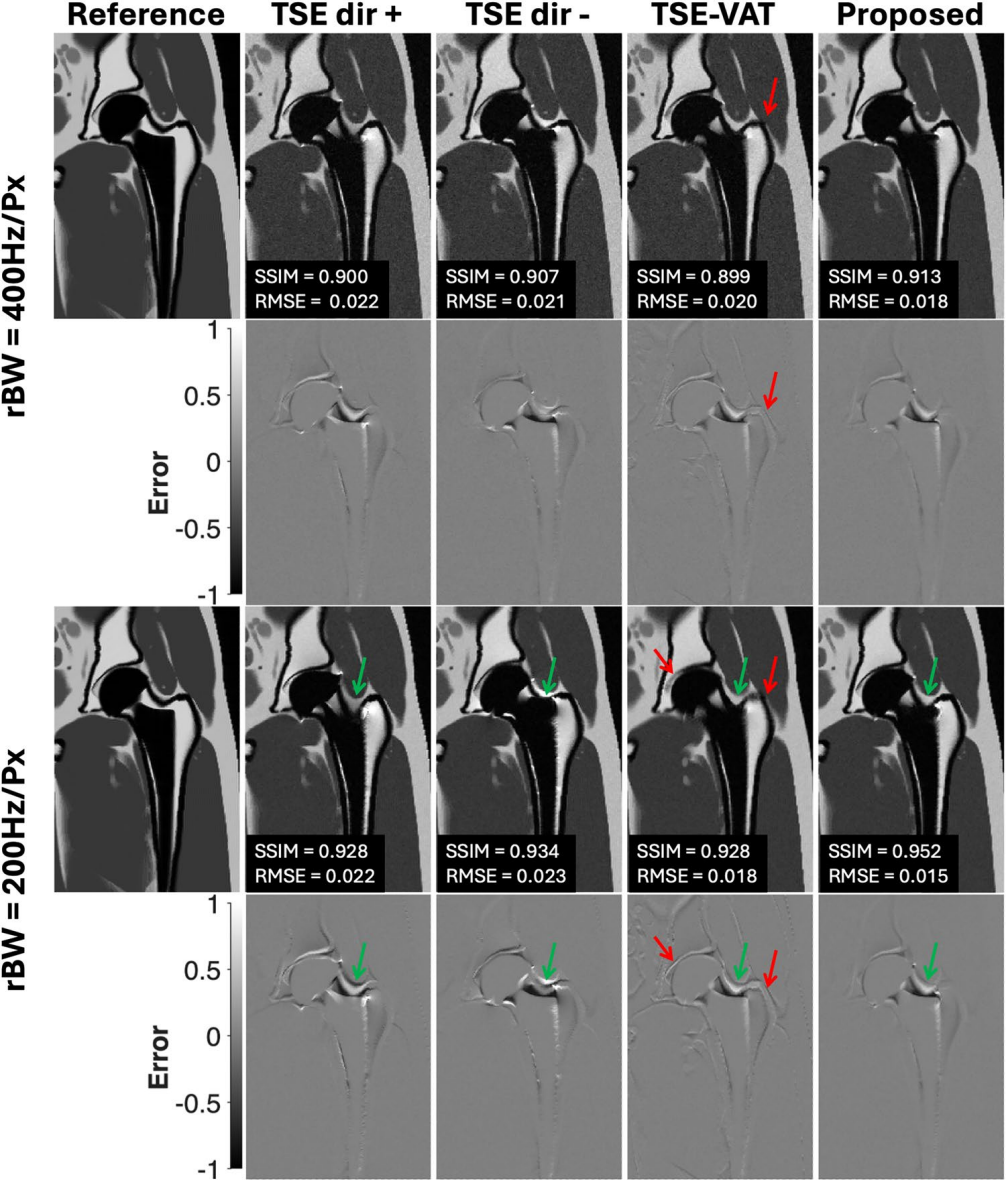
Simulation results comparing TSE, TSE-VAT, and the proposed RE-TSE reconstruction against a noiseless reference image. Rows 1–2 correspond to a readout bandwidth of 401 Hz/Px, and rows 3–4 correspond to 200 Hz/Px. All images were normalized by the maximum intensity of the reference image. For each bandwidth, reconstructed images from TSE with + RO direction, TSE with –RO direction, TSE-VAT, and the proposed method are shown alongside their corresponding error maps (difference from the reference). Structural similarity (SSIM) and root mean square error (RMSE) values are labeled. The proposed method yields the highest SSIM and lowest RMSE, indicating improved fidelity to the ground-truth reference, especially at low bandwidth. While VAT reduces geometric distortion, it also introduces substantial blurring, resulting in clear structural loss (highlighted with red arrows). The proposed reconstruction demonstrates comparable ability to TSE-VAT in correcting Δƒ-induced artifacts near the implant neck (green arrows), while better preserving spatial detail

Figure 3 presents the simulation results comparing TSE, TSE-VAT, and the proposed model-based reconstruction, with the noiseless image without off-resonance as ground truth. At a high readout bandwidth (401 Hz/Px), both methods exhibit reduced distortion, though differences remain visible: TSE-VAT introduces blurring, whereas the proposed method achieves the highest structural fidelity, as reflected by the highest SSIM (0.913 vs. 0.899 for VAT) and lowest RMSE (0.018 vs. 0.020). At a low bandwidth (200 Hz/Px), where Δƒ-induced displacement is more severe, TSE shows substantial distortion, and TSE-VAT corrects displacement but suffers from pronounced blurring and structural loss, clearly visible in both the image and the error map (red arrows). The proposed reconstruction achieves the highest SSIM and lowest RMSE, and substantially reduces residual errors while preserving anatomical details. Notably, artifacts near the implant head are strongly reduced (green arrows), with performance comparable to TSE-VAT. Taken together, these simulations show that the proposed method achieves off-resonance artifact correction comparable to VAT while maintaining superior spatial resolution, particularly in the low-bandwidth regime where distortion correction is most challenging.

**Fig. 4:**
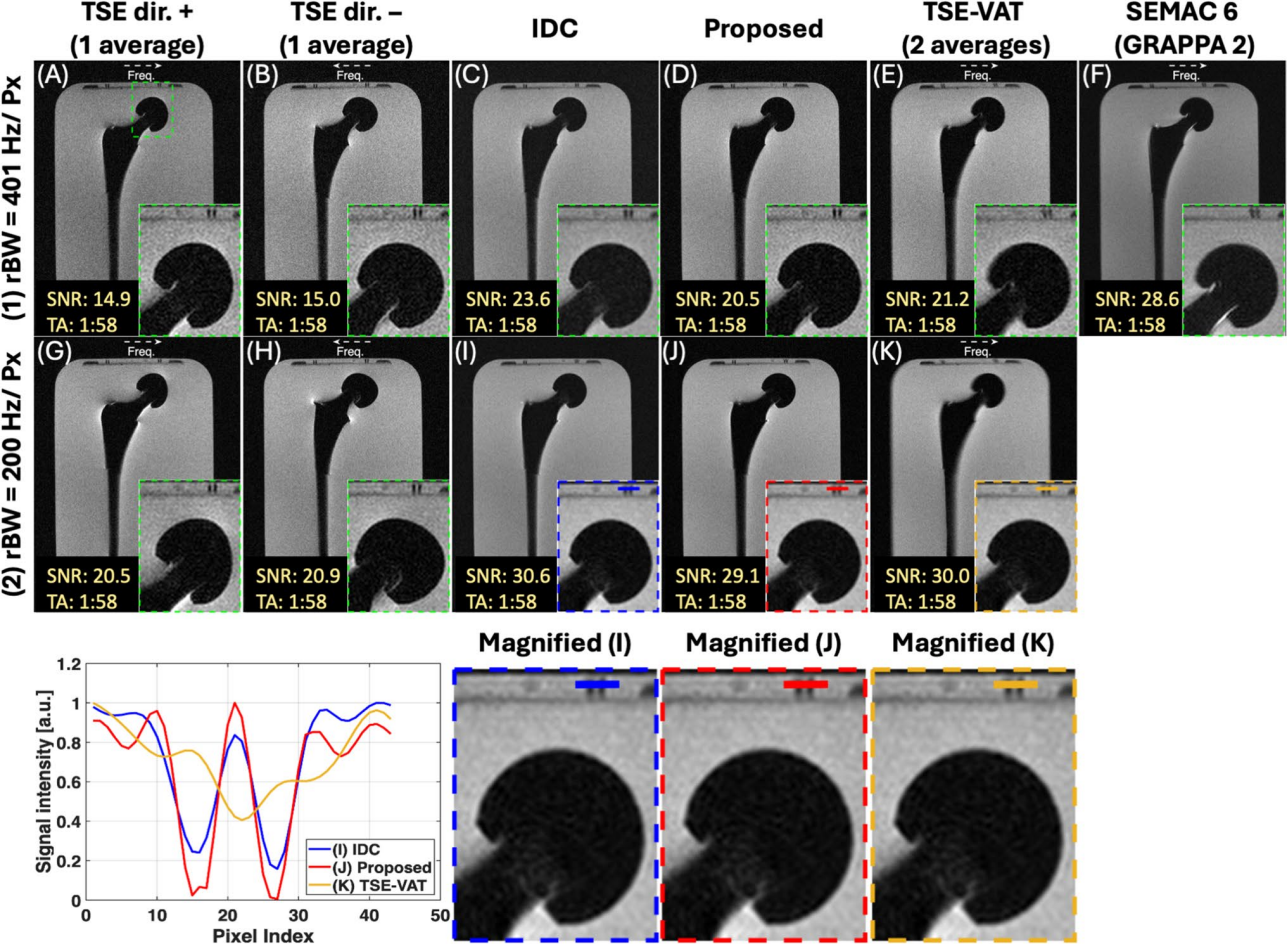
Hip implant phantom with a ceramic head and titanium stem. Frequency-encoding direction is labeled by white arrows. aSNR and acquisition time (**A**) are shown in the lower left of each image. Row **(1)**: high readout bandwidth (401 Hz/Px); row (**2**): low readout bandwidth (200 Hz/Px). Image-domain displacement correction (IDC), the proposed method RE-TSE, TSE-VAT, and SEMAC correct distortion near the acetabular cup in both high- and low-bandwidth images (zoomed-in boxes). Low bandwidth yields ~ 1.6 × higher aSNR than high bandwidth. The third row: line profile along the plastic grid for low-bandwidth case, comparing signal transition among **I** IDC, **J** the proposed method and **K** TSE-VAT

Figure 4 illustrates the performance of the proposed method using GNL and estimated displacement maps from the reverse-encoding image in a hip implant phantom. All images are GNL-corrected. Compared to conventional TSE images, the proposed method, TSE-VAT, and SEMAC effectively correct in-plane distortion at both 400 Hz/Px and 200 Hz/Px readout bandwidths. The sharper line profiles (bottom row, 200 Hz/Px) along the plastic insert demonstrate that the proposed reconstruction reduces the smoothing effect observed in the image-domain correction (IDC) and substantially mitigates the severe blurring seen in TSE-VAT. The reconstruction times for IDC and the proposed method were approximately 3.8 s and 7.6 s per slice, respectively. The aSNR of images acquired at 200 Hz/Px is approximately 1.4 × higher than that of 400 Hz/Px, consistent with the expected inverse square-root dependence of SNR on readout bandwidth.

**Fig. 5:**
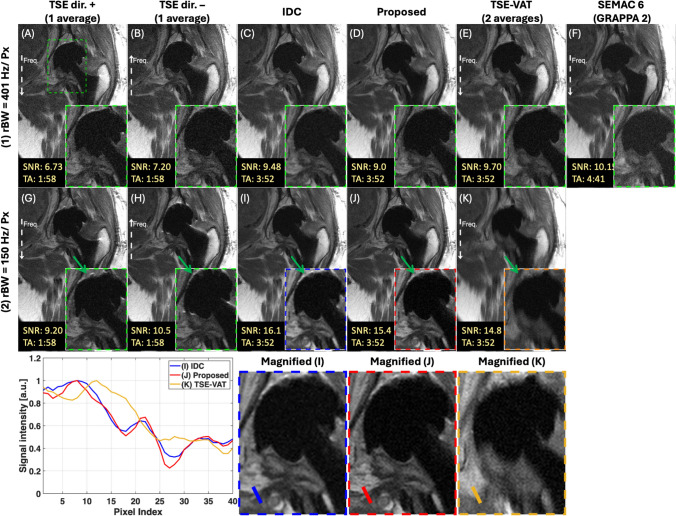
MR images of a 74-year-old female (BMI of 23.5 kg/m^2^) of left THA (titanium). aSNR and acquisition times (TA) are indicated in yellow in the lower-left corner. Frequency-encoding direction is labeled by white arrows. Opposite distortions are observed in the TSE acquisition pairs in (**A**)(**B**) and (**G**)(**H**). The zoomed-in images show that IDC, the proposed method, TSE-VAT and SEMAC correct in-plane distortion near the acetabular cup (green arrows). **E**, **F**, **K** Blurring is evident in TSE-VAT and SEMAC at both high and low bandwidths. Line profiles for the 120 Hz/Px case are drawn on small structures to compare signal transitions and smoothness among IDC, the proposed method, and TSE-VAT

Figure 5 shows representative results from a patient with total hip arthroplasty. Across both high and low readout bandwidths, the proposed model-based RE-TSE reconstruction effectively corrects in-plane distortion near the acetabular cup, consistent with IDC, TSE-VAT, and SEMAC. At low bandwidth (150 Hz/Px), the proposed method achieves substantially higher aSNR (~ 1.63 × under the current protocol) while avoiding the increased blurring seen in TSE-VAT and the smoothing introduced by IDC, enabling clearer depiction of fine peri-prosthetic structures. The line profiles, shown in the bottom row for the 150 Hz/Px case, demonstrate that the proposed method preserves sharper transitions compared to IDC and TSE-VAT. The reconstruction times for IDC and the proposed method were approximately 4.0 s and 8.4 s per slice, respectively. Results from two more THA patients are provided in Supplementary Information Fig. S1 and S2.

**Fig. 6:**
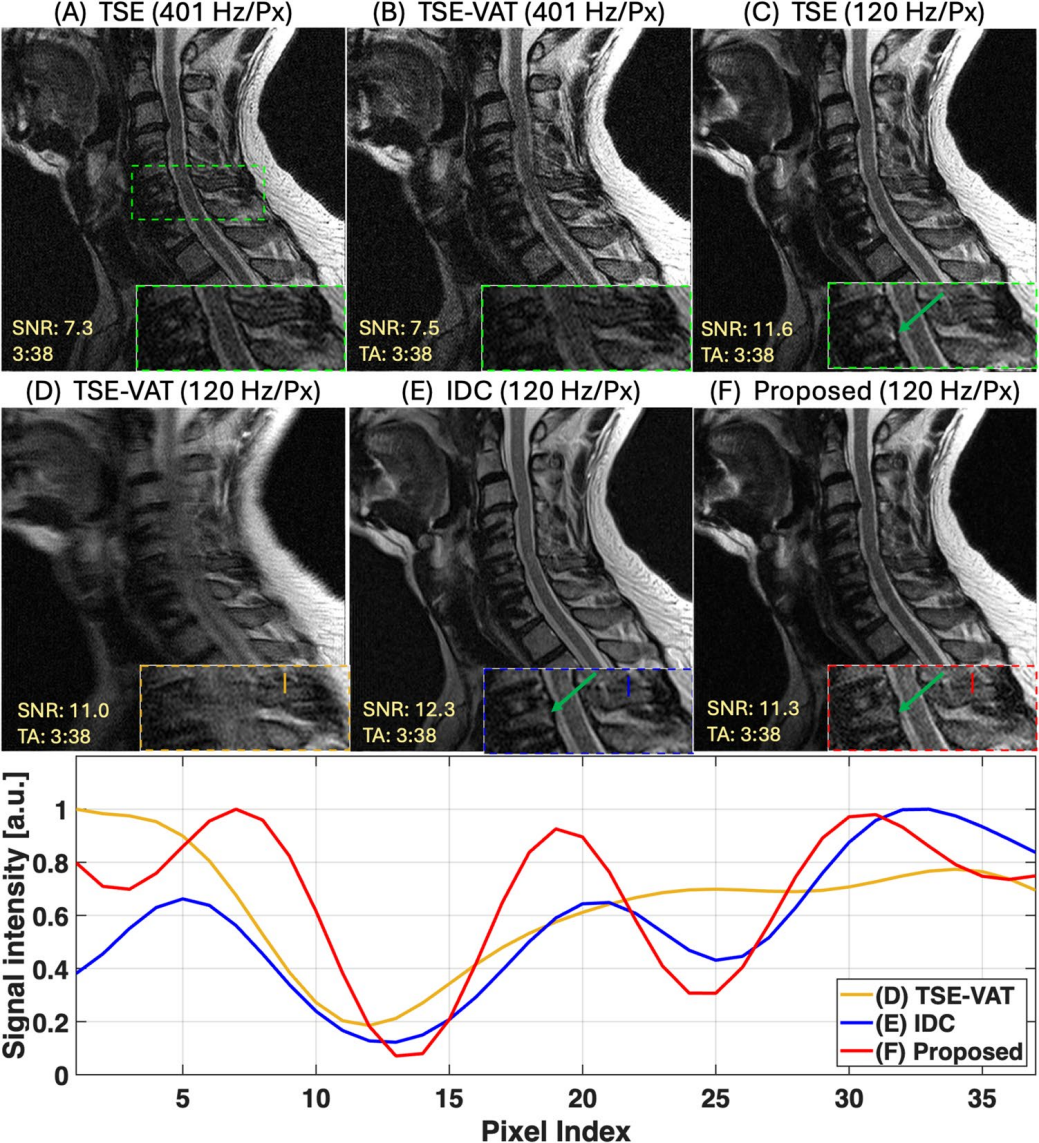
T2-weighted images of a 59-year-old male (BMI of 23.1 kg/m^2^) with anterior cervical discectomy and fusion (titanium). aSNR and acquisition time (TA) are labeled in the lower-left corner. Frequency-encoding direction is labeled by white arrows. **A** High-bandwidth TSE with less distortion but low aSNR; **B** TSE-VAT at 401 Hz/Px shows blurring and low aSNR; **C** low bandwidth (120 Hz/Px) TSE with higher aSNR but larger distortion and signal pile-up (green arrows); **D** low-bandwidth (120 Hz/Px) TSE-VAT shows severe blurring; **E** IDC and **F** the proposed method applied to low-bandwidth RE-TSE reduce distortion around the screws (green arrows) while maintaining high aSNR. Line profiles for the low-bandwidth case are shown to compare the degree of smoothing across methods

Figure 6 shows results from the patient with anterior cervical discectomy and fusion. Using a low readout bandwidth (120 Hz/Px), the TSE acquisition in (C) achieves higher aSNR (~ 1.60 ×) compared to the high-bandwidth TSE in (A), given the same echo train length and number of averages. However, this low-bandwidth TSE image exhibits strong Δƒ-related distortion, including signal pile-up near the screws (arrows). After correction, the screws are clearly delineated and the signal pile-up is removed, as demonstrated in (E) for IDC and (F) for the proposed method, with both producing comparable distortion correction. In contrast, TSE-VAT in (B) and (D) fails to preserve anatomical detail because of substantial blurring and reduced SNR. The line profile from the low-bandwidth cases further shows that the proposed method, with sharper transition, preserves local signal variations with less smoothing compared to IDC and TSE-VAT. The reconstruction times for IDC and the proposed method were approximately 4.9 s and 9.1 s per slice, respectively.

## Discussion

We present a model-based artifact correction near metal at 0.55 T, enabling the use of low readout bandwidth to improve both SNR and spatial resolution. This method utilizes TSE acquisitions with opposite frequency-encoding directions to reconstruct a single distortion-corrected image through iterative reconstruction, incorporating GNL and off-resonance displacement maps. This approach reduces displacement artifacts along the frequency-encoding direction induced by metal implants. Its feasibility was tested in both phantom studies and in vivo subjects, indicating its potential use for increased SNR in metal MRI at 0.55 T without increasing scan time. The improved SNR may significantly benefit hip and cervical spine imaging. The preserved high resolution after distortion correction validated in phantom and in vivo studies highlights the advantage of a model-based image reconstruction integrating displacement information compared to conventional correction methods. The feasibility of the phase-constrained Type-1 NUFFT model was supported by its agreement with ∆ƒ-based image-domain correction, and this model can be extended to partial Fourier reconstruction, as the two reversed-encoding k-spaces provide complementary information [38].

Employing a lower readout bandwidth at 0.55 T enables higher SNR, but it also increases the severity of ∆ƒ-related distortion. Conventional VAT-based sequences reduce geometric distortion but introduce substantial blurring, particularly at low bandwidth. Image-domain correction (IDC) avoids VAT blurring and therefore preserves more detail but still exhibits smoothing. In contrast, the proposed model-based reconstruction produces the least blurring among the three approaches, enabling the SNR benefits of low bandwidth to be realized without compromising spatial resolution. Notably, higher bandwidths potentially enable shorter echo spacing, which can be leveraged to increase turbo factor and acquire additional signal averages within the same scan time, partially recovering SNR while reducing distortion. Optimization of this approach for 0.55 T THA imaging will benefit from a comprehensive exploration of the tradeoff between residual readout distortion and SNR, which remains to be the future work. We expect this will require experimental studies using a modular phantom [39], with a range of imaging readout bandwidth, turbo factor, and number of averages.

This study has limitations. First, we tested the proposed method on a small number of subjects, and larger numbers are needed to understand its broader applicability. In particular, it should be tested in subjects with a variety of titanium implant configurations, and a variety of body habitus, which impacts the baseline SNR. Second, we did not correct distortion in the slice-select direction [20] which is minimal for titanium-based implants at 0.55 T. Based on simulations (not shown due to space limitations) [35], less than 0.5% of spins within 2 cm of titanium THA at 0.55 T will experience slice-select shift ≥ 1 slice thickness for the sequences used in this study. This shift can be further reduced by increasing the RF excitation bandwidth, which is feasible at 0.55 T due to the relaxed SAR constraints. This work intentionally neglects slice-select distortion and targets frequency-encoding distortions, which tend to be more prominent and are exacerbated when low readout bandwidths are used to improve SNR. The proposed method is intended as a practical and efficient alternative to conventional TSE and TSE + VAT at 0.55 T, which still make up the majority of clinical protocols [40]. Third, phase errors in TSE, such as those from eddy currents and concomitant gradient fields, were not considered. If needed, these could be incorporated into the signal model. For example, the effects can be predicted using gradient impulse response functions (**GIRFs**) from phantom-based measurements, and modeled as spatially varying phase terms using the MaxGIRF framework [41–43]. Fourth, we chose to keep the echo train length and number of averages consistent among sequences compared. Imaging with a higher readout bandwidth would enable a longer echo train length while maintaining the same level of T2 blurring. This would also partially compensate for the lower aSNR in high-bandwidth images. In addition, this study focuses only on B_0_-related effects; B₁-related artifacts near metal [44] were not investigated, although prior work has shown that B₁-related artifacts are reduced at 0.55 T compared with 1.5 T [12].

## Conclusion

We demonstrate a novel approach to correct in-plane distortions caused by metal implants at 0.55 T that enables the use of low readout bandwidth, which enhances SNR while preserving resolution and maintaining scan times comparable to conventional approaches.

## Supplementary Information

Below is the link to the electronic supplementary material.

Supplementary file1 (DOCX 3101 KB)

## Data Availability

The code and sample data (ISMRMRD format) that support the findings of this study are openly available in GitHub at https://github.com/usc-mrel/RETSE_Metal.

## Abbreviations

ACR: American College of Radiology
ACDF: Anterior cervical discectomy and fusion
IDC: Image-domain correction
GNL: Gradient nonlinearity
MAVRIC-SL: Multi-acquisition with variable-resonances image combination selective
NUFFT: Non-uniform fast Fourier transform
PCG: Preconditioned conjugate gradient
RE-TSE: Reverse-encoding turbo spin echo
ROI: Region of interests
SE: Spin echo
SENSE: Sensitivity encoding
SEMAC: Slice encoding for metal artifact correction
SNR: Signal-to-noise ratio
SSIM: Structural similarity index
THA: Total hip arthroplasty
TSE: Turbo spin echo
VAT: View angle tilting
