# Supplementary material for: Distortion correction in TSE near titanium implants at 0.55 T using reversed frequency-encoding and model-based reconstruction
Source: MAGMA. 2025 Dec 22;39(4):593–604. doi: 10.1007/s10334-025-01315-6 (PMC13447556; doi:10.1007/s10334-025-01315-6)
Supplement: Supplementary file 1 [file 10334_2025_1315_MOESM1_ESM.docx]

**Supplementary Information Figure S1.** MR images of a 61-year-old female (BMI of 29kg/m^2^) of right THA (titanium). aSNR and acquistion times (TA) are indicated in yellow in the lower-left corner. Frequency-encoding direction is labeled by white arrows. Opposite distortions are observed in the TSE acquisition pairs in **(A)(B)** and **(G)(H).** The zoomed-in images show that IDC, the proposed method, TSE-VAT and SEMAC correct in-plane distortion near the acetabular cup. (**E)(F)(K)** Blurring is evident in TSE-VAT and SEMAC at both high and low bandwidths. Line profiles for the 120 Hz/Px case are drawn on small structures to compare signal transitions and smoothness among IDC, the proposed method, and TSE-VAT.

**Supplementary Information Figure S2.** The post-THA (titanium) subject was imaged at an in-plane resolution of 0.875 mm² (66-year-old male, BMI of 28.1kg/m^2^). aSNR and acquistion times (TA) are indicated in yellow in the lower-left corner. Frequency-encoding direction is labeled by white arrows. Opposite distortions are observed in the TSE acquisition pairs in **(A)(B)** and **(G)(H).** The zoomed-in images show that IDC, the proposed method, TSE-VAT and SEMAC correct in-plane distortion near the acetabular cup. (**E)(F)(K)** Blurring is evident in TSE-VAT and SEMAC at both high and low bandwidths. Line profiles for the 120 Hz/Px case are drawn on small structures to compare signal transitions and smoothness among IDC, the proposed method, and TSE-VAT.

**Supplementary Information Table S1.** Detailed imaging parameters for the hip implant phantom, scanned in the coronal plane. Note that the echo times (TE) are 37 ms for sequences with a bandwidth of 200 Hz/Px and 34 ms for those with 401 Hz/Px to obtain PD-weighted images**.** Scan time, 3:52, is for one bandwidth TSE experiment. RE-TSE is a pair of opposite readout TSE.

| Parameter | RE-TSE | TSE-VAT | SEMAC 6 |
| --- | --- | --- | --- |
| TE | 37 and 34 ms | 37 and 34 ms | 31 ms |
| TR | 2000 ms | 2000 ms | 2100 ms |
| Bandwidth | 200 and 401 Hz/Px | 200 and 401 Hz/Px | 401 Hz/Px |
| Slice thickness | 4 mm | 4 mm | 4 mm |
| Base resolution | 320 | 320 | 320 |
| FOV read | 280 mm | 280 mm | 280 mm |
| FOV phase | 100 % | 100 % | 100 % |
| Slices | 12 | 12 | 12 |
| Average | 1 for each direction | 2 | 1 |
| Turbo Factor | 8 | 8 | 11 |
| Echo Spacing | 9.34/6.86 ms | 9.34/6.86 ms | 7.82 ms |
| Parallel imaging | N/A | N/A | N/A |
| Scan time | 3:52 | 3:52 | 6:22 |

**Supplementary Information Table S2.** Detailed imaging parameters for THA subjects, scanned in the coronal plane. Note that the echo times (TE) are 33 ms for sequences with a bandwidth of 150 Hz/Px and 34 ms for those with 401 Hz/Px to obtain PD-weighted images**.** RE-TSE is a pair of opposite readout TSE

| Parameter | RE-TSE | TSE-VAT | SEMAC 6 |
| --- | --- | --- | --- |
| TE | 33 and 34 ms | 33 and 34 ms | 31 ms |
| TR | 2000 ms | 2000 ms | 2100 ms |
| Bandwidth | 150 and 401 Hz/Px | 150 and 401 Hz/Px | 401 Hz/Px |
| Slice thickness | 4 mm | 4 mm | 4 mm |
| Base resolution | 320 | 320 | 320 |
| FOV read | 280 mm | 280 mm | 280 mm |
| FOV phase | 81.3 % | 81.3 % | 81.3 % |
| Slices | 20 | 20 | 20 |
| Average | 1 for each direction | 2 | 1 |
| Turbo Factor | 8 | 8 | 11 |
| Echo Spacing | 11/6.86 | 11/6.86 | 7.82 |
| Parallel imaging | N/A | N/A | GRAPPA-2 |
| Scan time | 3:52 | 3:52 | 4:41 |

**Supplementary Information Table S3.** Detailed imaging parameters for in-vivo ACDF subject, scanned in the sagittal plane. Note that the echo times (TE) are 114 ms for sequences with a bandwidth of 120 Hz/Px and 110 ms for those with 401 Hz/Px to acquire T2-weighted images**.** RE-TSE is a pair of opposite readout TSE.

| Parameter | TSE | RE-TSE | TSE-VAT |
| --- | --- | --- | --- |
| TE | 114 and 110 ms | 114 ms | 110 ms |
| TR | 4460 ms | 4460 ms | 4460 ms |
| Bandwidth | 120 and 401 Hz/Px | 120 Hz/Px | 401 Hz/Px |
| Slice thickness | 4 mm | 4 mm | 4 mm |
| Base resolution | 320 | 320 | 320 |
| FOV read | 279 mm | 279 mm | 279 mm |
| FOV phase | 100 % | 100 % | 100 % |
| Slices | 19 | 19 | 19 |
| Average | 2 | 1 for each direction | 2 |
| Turbo Factor | 16 | 16 | 16 |
| Echo Spacing | 12.7/7.88 | 12.7/7.88 | 12.7/7.88 |
| Parallel imaging | N/A | N/A | N/A |
| Scan time | 3:38 | 3:38 | 3:38 |
